# Anti-Photoaging Effect of *Pinctada martensii* Hydrolysates on Ultraviolet B-Irradiated Nude Mice Skin

**DOI:** 10.3390/md24030097

**Published:** 2026-02-28

**Authors:** Mengfen Wei, Dongcheng Liu, Shiyuan Chang, Lijun You, Oliy Akhmedov

**Affiliations:** 1 School of Food Science and Engineering, South China University of Technology, Guangzhou 510640, China; weimengfen4006@163.com (M.W.); amsychang@163.com (S.C.); 2 State Key Laboratory of Advanced Papermaking and Paper-Based Materials, South China University of Technology, Guangzhou 510640, China; 3 Guangzhou Quality Testing and Inspection Institute, Guangzhou 511447, China; liudongcheng1996@163.com; 4 Institute of Bioorganic Chemistry of the Academy of Sciences of Uzbekistan, Tashkent 100125, Uzbekistan; akhmedov.oliy@gmail.com

**Keywords:** anti-photoaging, *Pinctada martensii* meat, protein enzymatic hydrolysates, differential metabolites, gut microbiota

## Abstract

*Pinctada martensii* is a marine resource with potential for bioactive peptide development, but its anti-photoaging properties remain underexplored. In this study, *Pinctada martensii* meat hydrolysates (PME) were prepared by enzymatic hydrolysis, and their anti-photoaging effects were evaluated in an in vivo ultraviolet-B (UVB)-irradiated nude mouse model. The results showed that PME markedly ameliorated UVB-induced skin damage. UVB increased epidermal thickness from 21.60 μm in the Control to 47.50 μm in the Model, and PME reduced epidermal thickness to 22.46 μm. Dermal collagen content decreased from 64.58% in the Control to 26.22% in the Model and was restored to 52.75% by PME. UVB upregulated matrix metalloproteinases-1 (MMP-1), MMP-3 and MMP-9 by approximately 2.20-, 1.93- and 3.09-fold relative to the Control, and PME suppressed these matrix metalloproteinases (MMPs) by approximately 61%, 65% and 52%, respectively. Extracellular signal-regulated kinase (ERK) expression increased to 1.41-fold in the Model and was reduced to about 1.05-fold after PME treatment, suggesting inhibition of collagen degradation-related pathways. Untargeted serum metabolomics identified 205 differential metabolites between the Model and the Control, and PME shifted metabolite profiles toward those of the Control. Total short-chain fatty acids (SCFAs) decreased from 868.69 μmol/g in the Control to 301.34 μmol/g in the Model and increased to approximately 562 μmol/g after PME treatment, accompanied by modulation of the gut microbiota including recovery of Lachnospiraceae members, indicating involvement of the gut–skin axis. These findings support the potential of *Pinctada martensii* meat as a source for developing novel functional foods targeting skin photoaging.

## 1. Introduction

Skin photoaging is driven by chronic ultraviolet (UV) exposure and is characterized by wrinkles formation, loss of skin elasticity, pigmentation disorders, and dryness [1]. The study at the molecular level revealed that UV irradiation upregulated the expression of MMPs, leading to extracellular matrix (ECM) degradation, which collectively resulted in structural, morphological, and functional deterioration of the skin [2]. Epidemiological evidence also links photoaging to skin conditions such as actinic elastosis, actinic keratosis, and melanoma, underscoring its public health relevance [3]. A variety of approaches have been employed to evaluate photoaging, including collagenase and elastase inhibition assays, MMP expression analysis, dermal collagen quantification, and skin barrier assessment. Nevertheless, increasing evidence indicates that the regulatory mechanisms underlying photoaging exhibit systemic characteristics, involving not only localized skin alterations but also systemic metabolic regulation and gut microbiota–host interactions [4,5], emphasizing the importance of investigating anti-photoaging interventions from a systemic perspective.

In recent years, natural bioactive compounds, including peptides [6], polyphenols [7], and polysaccharides [8], have emerged as preventive and therapeutic options for photoaging. Among these, protein hydrolysates have demonstrated significant biological activities in protecting against and repairing photoaged skin [9,10]. Xu et al. reported that walnut protein hydrolysates attenuated UV-induced photoaging through inhibiting the NF-κB/MMP-1 signaling pathway in female rats [11]. Peng et al. isolated anti-photoaging peptides from oyster (Crassostrea hongkongensis) enzymatic protein hydrolysates and demonstrated that these peptides modulate the MAPK/NF-κB signaling pathway in HaCaT cells to prevent photoaging [12].

*Pinctada martensii*, distributed along the coasts of mainland China and throughout the South China Sea, is primarily valued for pearl production. Its protein-rich meat has also drawn attention, highlighting its potential as a high-value marine shellfish resource [13,14]. However, due to the lack of deep processing technologies, most *Pinctada martensii* meat is discarded as a by-product after pearl harvesting, resulting in substantial resource waste [15]. Previous studies have demonstrated that hydrolysates derived from *Pinctada martensii* meat exhibited diverse bioactivities, including hypoglycemic effects [14], wound healing promotion [16], anti-inflammatory activity [17], and antioxidant activities [18]. Moreover, *Pinctada martensii* meat hydrolysates show potential anti-photoaging effects [19]. Nevertheless, the in vivo mechanisms underlying the anti-photoaging effects of *Pinctada martensii* meat hydrolysates remain largely unexplored.

Therefore, in this study, anti-photoaging hydrolysates from *Pinctada martensii* meat were prepared utilizing digestive enzymes from the gastrointestinal tract. The effect and mechanism of anti-photoaging hydrolysates were investigated using the UVB-induced BALB/c nude mice model. These findings have implications for enhancing the economic value of *Pinctada martensii* and for developing anti-photoaging products.

## 2. Results and Discussion

### 2.1. The Effects of PME on Body Weight in Photoaged Nude Mice

**Figure 1:**
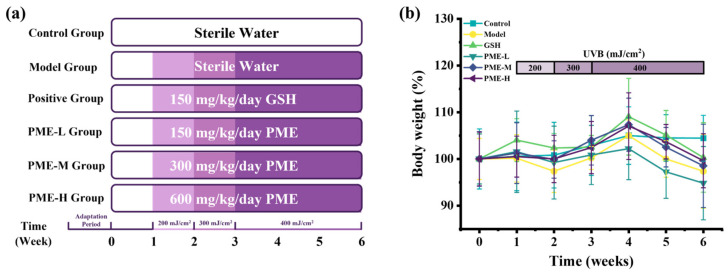
Schematic diagram of experimental procedure (**a**); effects of PME on the body weight of photoaged nude mice (**b**).

Body weight is an important indicator of health status and of potential sample toxicity in mice [20,21]. In this study, nude mice received PME at 150, 300, or 600 mg/kg/day (PME-L, PME-M, PME-H), and body weight was monitored. As shown in Figure 1b, the body weight of mice in the Control group gradually increased from week 0 to week 4 and remained relatively stable from week 4 to week 6. Mice in the Model, glutathione (GSH), PME-L, PME-M, and PME-H groups exhibited an initial decline in body weight followed by partial recovery during weeks 1–4 and a subsequent decrease during weeks 4–6. Compared with the Control group, body weight was lower in the Model group, consistent with UVB-induced physiological stress. Treatment with GSH, PME-M, and PME-H effectively alleviated UVB-induced weight loss, whereas PME-L showed a limited effect on body weight recovery. This pattern is consistent with systemic physiological stress induced by repeated and progressively increased UVB dosing, which can alter appetite, energy metabolism and inflammatory status [22]. The ability of PME-M and PME-H to mitigate weight loss suggests a systemic protective effect potentially via anti-inflammatory, antioxidant or metabolic regulatory actions.

### 2.2. Macroscopic Evaluation in Photoaging Skin

**Figure 2:**
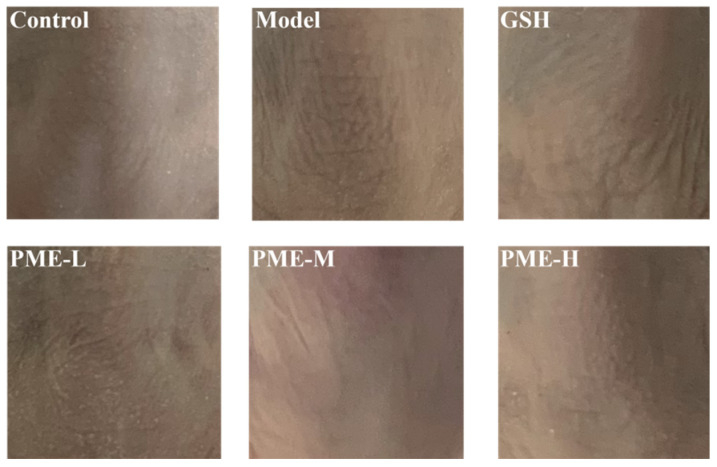
Effects of PME on the epidermal morphology of photoaged nude mice skin. Images were captured using a smartphone camera under consistent lighting conditions for gross morphological assessment. Control mice showed smooth dorsal skin with minimal fine wrinkles, whereas UVB exposure induced deep, coarse wrinkles and marked roughness. Oral GSH and all PME alleviated these changes.

Chronic UV exposure produces the typical clinical features of photoaging such as deepened wrinkles and increased skin roughness [23]. As shown in Figure 2, Control mice maintained smooth dorsal skin with only a few thin and shallow wrinkles, whereas UVB-exposed mice developed significant deep and thick wrinkles, along with marked skin roughness. Oral GSH and all three PME doses visibly ameliorated these macroscopic signs. Notably, among PME doses, the PME-M group produced the greatest improvement in macroscopic appearance. This observation aligns with previous reports showing that combined UVA/UVB exposure deepens dorsal transverse wrinkles, whereas oral administration of oyster hydrolysate smooths the skin in irradiated mice [24].

Previous studies have reported that enzymatic hydrolysates of *Pinctada martensii* meat are rich in leucine, asparagine, and glutamic acid [25,26,27]. Increasing evidence suggested that peptides containing these amino acid residues might exert skin-protective effects. For example, oral administration of glycine-leucine dipeptides could improve skin hydration and elasticity in UVB-irradiated hairless mice [28]. Similarly, glutamic acid-rich peptide extracts from tempeh showed protective effects against photoaging [29]. Therefore, the anti-photoaging activity observed in PME may be partially attributed to the specific amino acid composition of *Pinctada martensii* meat hydrolysates, particularly peptides enriched in leucine and glutamic acid.

### 2.3. The Effects of PME on Organ Indexes in Photoaged Nude Mice

**Figure 3:**
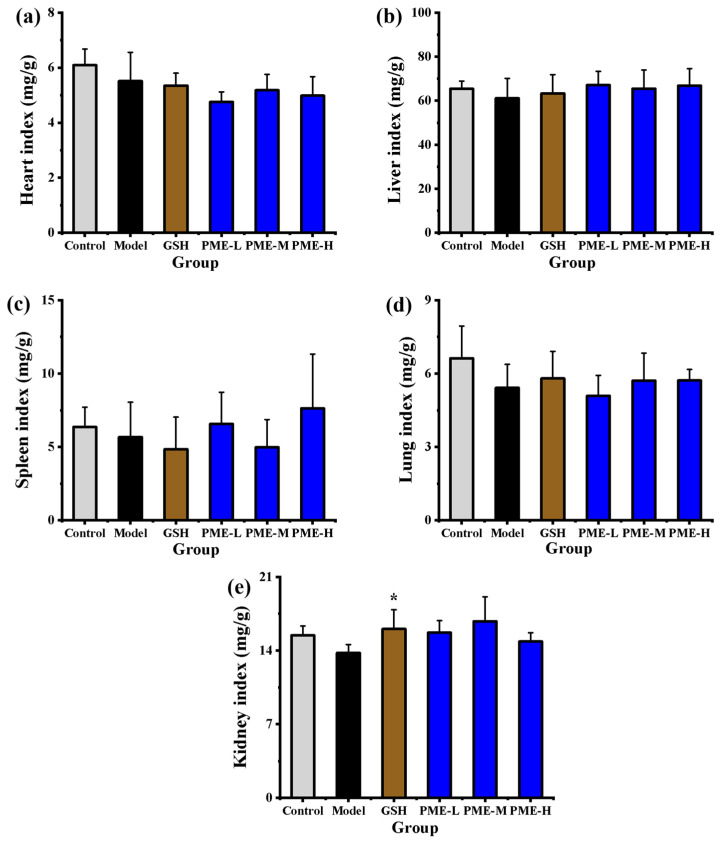
Effects of PME on heart index (**a**), liver index (**b**), spleen index (**c**), lung index (**d**), and kidney index (**e**) of photoaged nude mice. * *p* < 0.05 vs. Model group. Data are presented as the mean ± SD (*n* = 6).

Organ indices, defined as organ weight normalized to body weight, are commonly used to screen for developmental effects and systemic toxicity of drugs and bioactive substances [30]. As shown in Figure 3a–e, compared with the Control group, the heart, liver, spleen, lung, and kidney indexes in the Model group decreased by 9.57%, 6.60%, 10.85%, 18.22%, and 10.81%, respectively, but none of these differences reached statistical significance (*p* > 0.05), suggesting that the UVB exposure did not produce detectable changes in organ index under the present conditions. Treatment with PME at any dose did not produce significant changes in these organ indices relative to the Model group (*p* > 0.05), consistent with an absence of detectable organ toxicity in this study. Notably, oral GSH treatment produced a significant increase in the kidney index compared with the Model group (*p* < 0.05). Taken together, the organ-index data suggest that PME is well tolerated in photoaged nude mice at the tested doses and time frame, supporting further mechanistic and efficacy studies. Consistent with these findings, oral administration of protein hydrolysates from silver carp likewise produced no significant alterations in spleen, liver or kidney indexes in UVB-exposed nude mice [31].

### 2.4. The Effects of PME on Pathological Architecture in Photoaged Nude Mice

**Figure 4:**
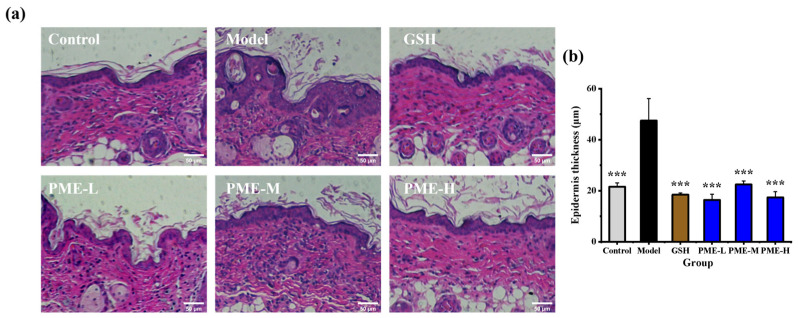
Effects of PME on the pathological architecture of photoaged nude mice skin. HE staining for epidermis and dermis in mice dorsal skin (**a**), epidermis thickness (**b**). Scale bar = 50 μm. *** *p* < 0.001 vs. Model group. Data are presented as the mean ± SD (*n* = 6).

The skin is composed of the epidermis and the underlying dermis. The epidermis is a stratified epithelium, containing one layer of proliferative cells and several layers of differentiated cells [32]. Ultraviolet exposure has been reported to induce an increase in epidermal thickness [33]. The pathological architecture was measured by HE staining to investigate the in vivo anti-photoaging activity of PME. As shown in Figure 4a,b, skin from the Control mice appeared healthy, with clearly defined epidermis, dermis, and collagen fibers. The epidermal layer exhibited uniform thickness, and the cells were densely and orderly arranged. After UVB irradiation, epidermal thickness in the Model group increased significantly to 47.50 μm (*p* < 0.05) versus the Control (21.60 μm). In addition, epidermal nuclei became irregular and dermal collagen fibers were fragmented and loosened. Under PME treatment, epidermal cell organization was restored, and the epidermal thickness decreased to 16.39 μm, 22.46 μm, and 17.40 μm after PME-L, PME-M, and PME-H, respectively, all significantly lower than the Model group (*p* < 0.05). Moreover, PME significantly (*p* < 0.05) reduced UVB-induced epidermal thickening to Control levels, with effects comparable to those of the positive control GSH (18.46 μm). For comparison, a previous study reported that UVB increased epidermal thickness from 6.70 μm in normal mice to 35.64 μm, and that treatment with mackerel scad skin collagen hydrolysate reduced thickness to 10.74 μm [34]. These findings indicate that UVB irradiation markedly increases epidermal thickness and disrupts dermal collagen, whereas PME mitigates these histopathological changes, supporting its anti-photoaging effect.

### 2.5. The Effects of PME on Collagen Fibers in Photoaged Nude Mice

**Figure 5:**
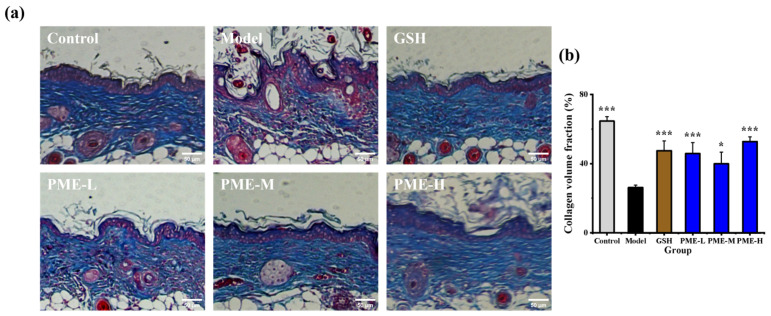
Effects of PME on collagen fibers of photoaged nude mice skin. Masson staining for epidermis and dermis in mice dorsal skin (**a**), collagen volume fraction (**b**). Scale bar = 50 μm. * *p* < 0.05 or *** *p* < 0.001 vs. Model group. Data are presented as the mean ± SD (*n* = 6).

Collagen fibers serve as the primary structural framework of the skin, providing stability and tensile strength [35]. Collagen accounts for approximately 75% of dermal dry weight [36]. UVB exposure disrupts dermal integrity by inducing collagen fiber degradation and loss, a feature of photoaging [37]. As shown in Figure 5a, collagen fibers in the Control group exhibit a well-organized arrangement with normal morphology. UVB irradiation significantly reduced collagen fiber content in the Model group (*p* < 0.05), accompanied by fiber fragmentation and disordered arrangement. Treatment with GSH or PME significantly increased collagen content (*p* < 0.05), resulting in denser, better-organized fibers with less fragmentation and reduced sparsity. As shown in Figure 5b, the collagen volume fraction in the skin was significantly reduced in the Model group (26.22%) compared with the Control group (64.58%, *p* < 0.05), indicating UVB-induced collagen loss. Treatment with PME-L, PME-M, and PME-H significantly (*p* < 0.05) increased the collagen volume fraction to 45.84%, 39.90%, and 52.75%, respectively. Moreover, there were no significant differences in collagen volume fraction between the PME-treated groups and the GSH group (47.48%), suggesting that PME effectively restored collagen levels comparable to GSH treatment. These results are consistent with previous reports showing that UV exposure reduces dermal collagen content and that antioxidants or protective treatments can mitigate collagen degradation. For example, Liu et al. [38] reported that oral administration of sea bass scales collagen hydrolysates restored collagen organization and increased collagen volume fraction in UV-irradiated mice, while Fan et al. [39] observed partial recovery of dermal collagen following collagen-EGCG combination treatment. Compared with these studies, the data in this study demonstrate that PME (particularly PME-H) restored collagen volume fraction to levels comparable with GSH in a nude mouse UVB model. These results demonstrate that UVB irradiation fragments and reduces dermal collagen fibers, whereas PME intervention attenuates this damage, highlighting its potent anti-photoaging effect.

### 2.6. The Effects of PME on Type I and Type III Collagens in Photoaged Nude Mice

**Figure 6:**
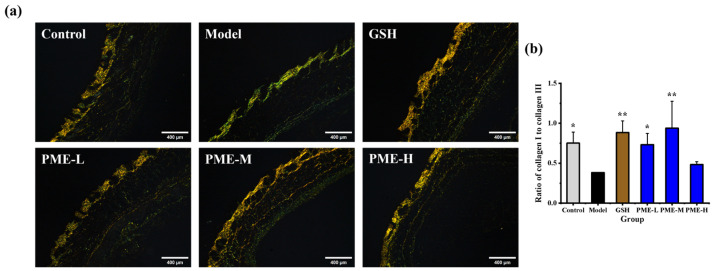
Effects of PME on type I and type III collagens of photoaged nude mice skin. Sirius Red staining of the dorsal skin (collagen I in red, collagen III in green) (**a**), ratio of collagen I to collagen III (**b**). Scale bar = 400 μm. * *p* < 0.05 or ** *p* < 0.01 vs. Model group. Data are presented as the mean ± SD (*n* = 6).

Type I collagen and type III collagen are the two predominant collagen types in the skin, accounting for approximately 70% and 15% of total collagen, respectively [36,40]. Type I collagen is a structural protein that provides tensile strength and elasticity, while type III collagen is a more relaxed collagen protein that contributes to skin softness and smoothness [41]. UVB irradiation induces degradation of type I collagen and upregulates the synthesis of type III collagen in the skin, leading to altered collagen composition [42]. In this study, the contents of type I and type III collagens in UVB-irradiated skin were measured by Sirius Red staining to assess the in vivo anti-photoaging effects of PME. As shown in Figure 6a,b, UVB irradiation reduced the type I/type III collagen ratio in the Model group by 49% compared with the Control group (*p* < 0.05). Treatment with GSH increased the collagen I/III ratio 2.32-fold relative to the Model group (*p* < 0.05). PME-L and PME-M increased the ratio 1.92-fold and 2.46-fold, respectively, restoring it to levels comparable with the Control group (*p* > 0.05). Many prior photoaging intervention studies focus on procollagen I as the primary outcome and therefore do not address whether treatments normalize the relative abundance of collagen subtypes [43,44]. By calculating the I/III ratio, the present data provides more comprehensive evidence that PME-M not only stimulates type I production but also re-establishes collagen subtype balance. These results suggest that PME can restore the type I/type III balance by reducing type I degradation and/or limiting type III synthesis, thereby supporting skin structure and function.

### 2.7. The Effects of PME on Levels of HYP, MMP-1, MMP-3 and MMP-9 in Photoaged Nude Mice

**Figure 7:**
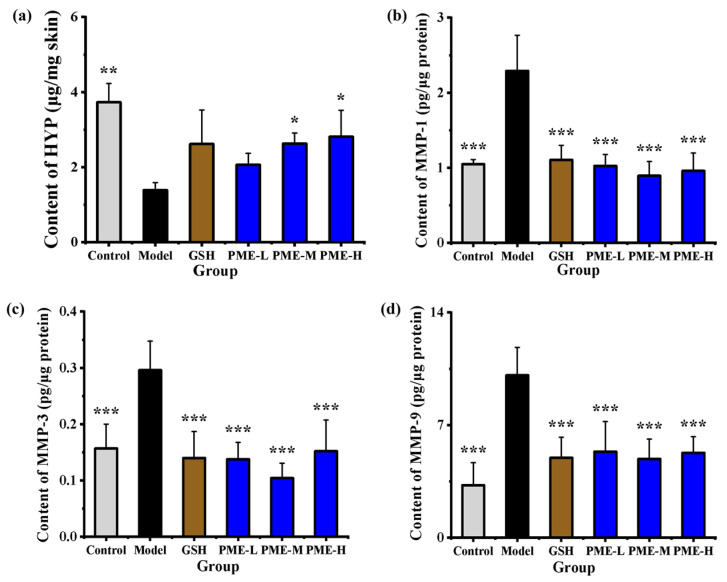
Effects of PME on contents of HYP (**a**), MMP-1 (**b**), MMP-3 (**c**), and MMP-9 (**d**) in photoaged nude mice skin. * *p* < 0.05, ** *p* < 0.01 or *** *p* < 0.001 vs. Model group. Data are presented as the mean ± SD (*n* = 6).

Hydroxyproline (HYP) is the main water-interacting amino acid in collagen. Its reduction impairs water bridges and alters collagen conformation [45,46]. Studies have shown that HYP content is reduced in photoaged skin [47], and thus HYP level serves as an index of collagen integrity in photoaging. MMPs are zinc-containing endopeptidases that degrade extracellular matrix (ECM) proteins such as collagen, fibronectin, elastin, and proteoglycans, contributing to photoaging [48]. UV-irradiated skin shows increased levels and activity of MMPs, notably MMP-1, MMP-3, and MMP-9 [49]. Their tissue levels reflect the extent of ECM degradation. In this study, the contents of HYP, MMP-1, MMP-3 and MMP-9 of UVB-irradiated nude mice were measured to investigate the in vivo anti-photoaging effects of PME. As shown in Figure 7a, skin HYP in the Model group was significantly lower than in the Control group (*p* < 0.05), decreasing by 62.82%, consistent with UVB-induced collagen disruption. Although treatment with GSH and PME increased HYP, but there was no significant difference compared with the Model group. Figure 7b–d show that MMP-1, MMP-3 and MMP-9 were significantly elevated in the Model group relative to the Control (*p* < 0.05), by 2.20-, 1.93- and 3.09-fold, respectively, indicating UVB-activated ECM degradation. Treatment with GSH and all PME doses significantly reduced MMP-1, MMP-3 and MMP-9 compared with the Model group (*p* < 0.05), restoring levels toward those of the Control and matching the positive control. PME-M produced the greatest inhibition, lowering MMP-1, MMP-3 and MMP-9 by 60.97%, 64.75% and 51.55%, respectively.

The significant suppression of MMPs without significant restoration of total HYP can be explained by several factors. MMPs expression and activation are rapid, early responses to UVB, whereas restoration of tissue collagen (reflected by total HYP) requires slower processes of collagen synthesis and matrix remodeling [50]. Collagen that has already been degraded and removed from the tissue cannot be rapidly replaced if fibroblast-mediated synthesis is not concurrently upregulated. These results indicated that UVB irradiation might disrupt collagen conformation by accelerating ECM degradation through the upregulation of MMP-1, MMP-3, and MMP-9. PME treatment effectively counteracted these UVB-induced alterations, with inhibitory effects on MMPs.

### 2.8. The Effects of PME on the Expression of ERK in Photoaged Nude Mice

**Figure 8:**
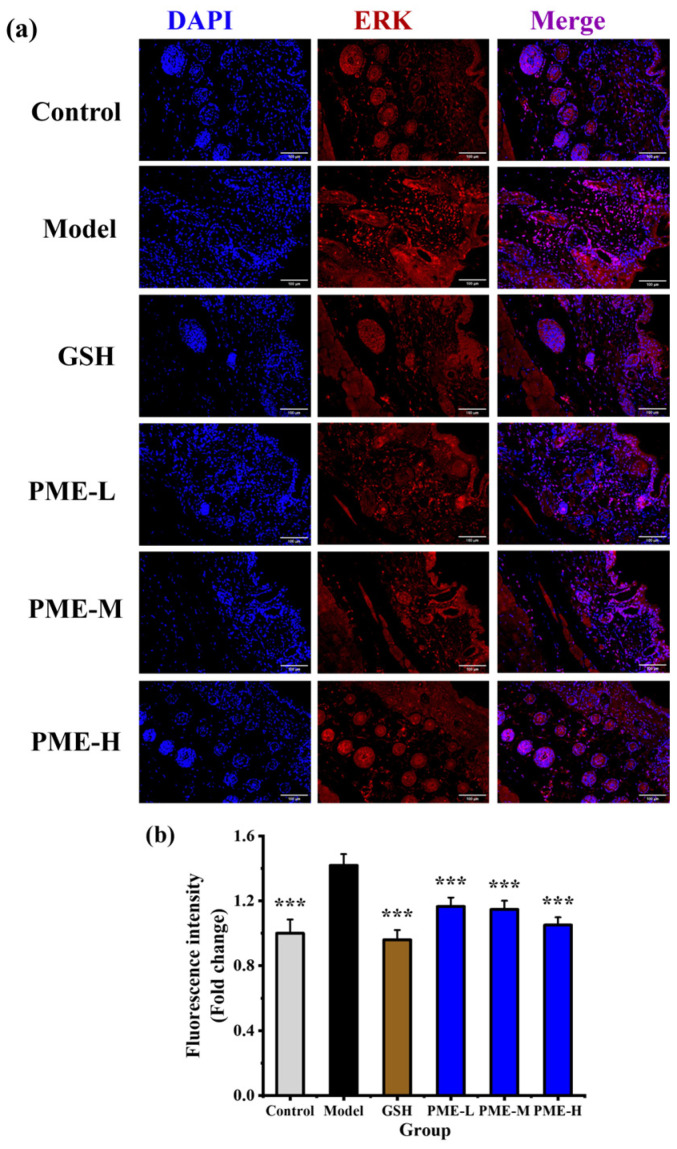
Effects of PME on expression of ERK in photoaged nude mice skin. Immunofluorescence staining of ERK protein (**a**), fluorescence intensity (**b**). Scale bar = 100 μm, *** *p* < 0.001 vs. Model group. Data are presented as the mean ± SD of three independent experiments.

Extracellular signal-regulated kinase (ERK), a key member of the MAPK family, has been identified as an important target for the chemoprevention of skin diseases [51]. UVB irradiation has been shown to activate ERK [52], and ERK expression is widely used as an indicator of photoaging. In this study, ERK expression in UVB-irradiated nude mouse skin was assessed by immunofluorescence staining to evaluate the in vivo anti-photoaging effect of PME. As shown in Figure 8a,b, ERK expression was significantly increased in the Model group compared with the Control group (*p* < 0.05), reaching 1.41-fold of the Control. PME treatment reduced ERK expression in a concentration-dependent manner, with values of 1.16-, 1.14- and 1.05-fold of the Control under PME-L, PME-M and PME-H, respectively. The ERK level in the PME-H group did not differ significantly from those in the GSH and Control groups (*p* > 0.05), indicating that PME-H inhibited ERK expression to an extent comparable with GSH and effectively restored ERK to control levels. However, ERK pathway activity is most reliably assessed by phosphorylated ERK (p-ERK), which reflects pathway activation. Measuring only total ERK by immunofluorescence cannot fully explain activation status or functional changes, and therefore the mechanistic conclusions regarding ERK should be interpreted with caution which is the limitation of the present study.

### 2.9. The Effects of PME on Serum Metabolomics in Photoaged Nude Mice

**Figure 9:**
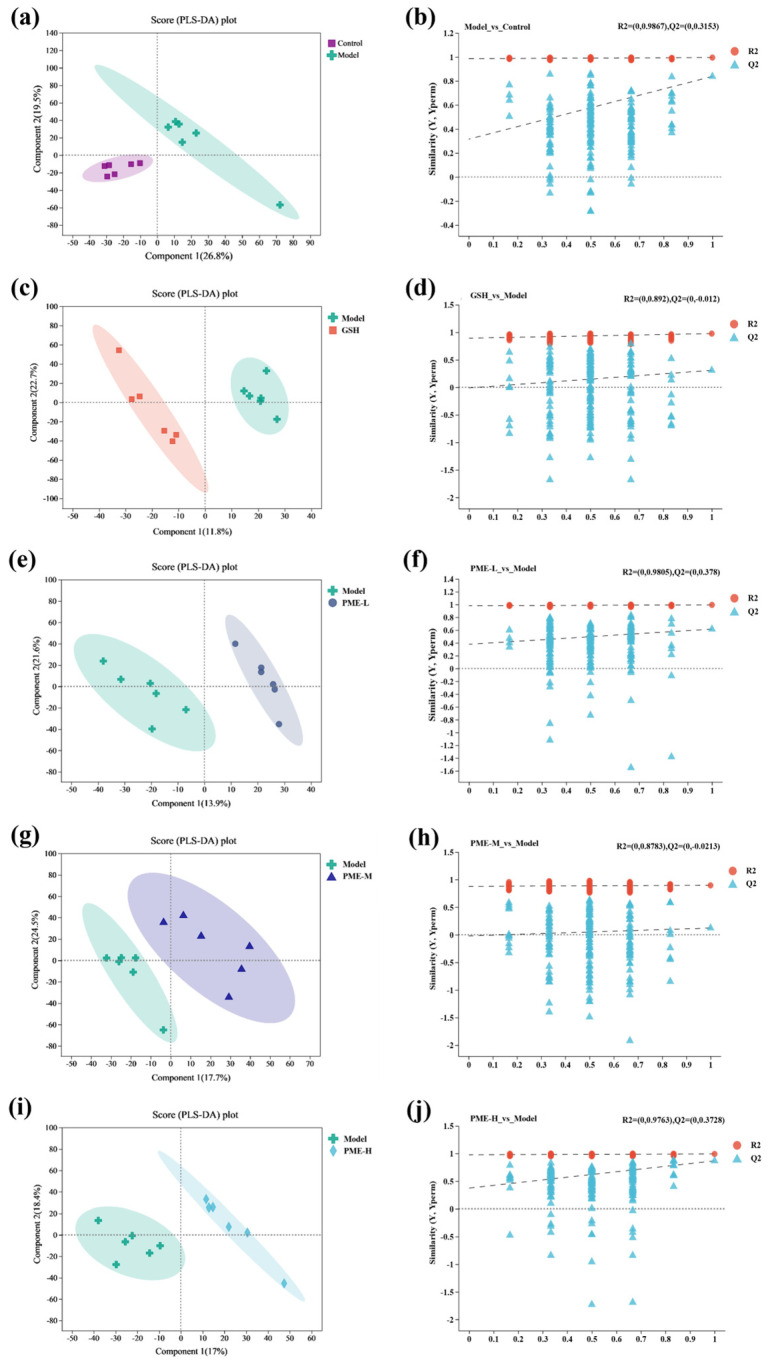
The changes of serum metabolisms in photoaging mice. PLS-DA scores plot between Model group and Control group (**a**), arrangement verification diagram between Model group and Control group (**b**), PLS-DA scores plot between GSH group and Model group (**c**), arrangement verification diagram between GSH group and Model group (**d**), PLS-DA scores plot between PME-L group and Model group (**e**), arrangement verification diagram between PME-L group and Model group (**f**), PLS-DA scores plot between PME-M group and Model group (**g**), arrangement verification diagram between PME-M group and Model group (**h**), PLS-DA scores plot between PME-H group and Model group (**i**), arrangement verification diagram between PME-H group and Model group (**j**).

PLS-DA was used to assess group separation, and model robustness was evaluated by R2 and Q2 values from permutation tests, with a Q2 intercept below 0.05 or an upward trend of the Q2 regression line taken as evidence of model reliability. As shown in Figure 9a,c,e,g,i, the Control and Model groups were clearly separated, showing R2Y of 0.9867 and Q2Y of 0.3153. All treatment groups were distinctly discriminated from the Model group, showing R2Y of 0.892 and Q2Y of −0.012 in the GSH and Model groups, R2Y of 0.9805 and Q2Y of 0.378 in the PME-L and Model groups, R2Y of 0.8783 and Q2Y of −0.0213 in the PME-M and Model groups, and R2Y of 0.9763 and Q2Y of 0.3728 in the PME-H and Model groups. As shown in Figure 9b,d,f,h,j, in all differential groups, R2Y values exceeded Q2Y values, and the Q2 regression lines exhibited an upward trend, confirming the robustness and reliability of the PLS-DA models.

**Figure 10:**
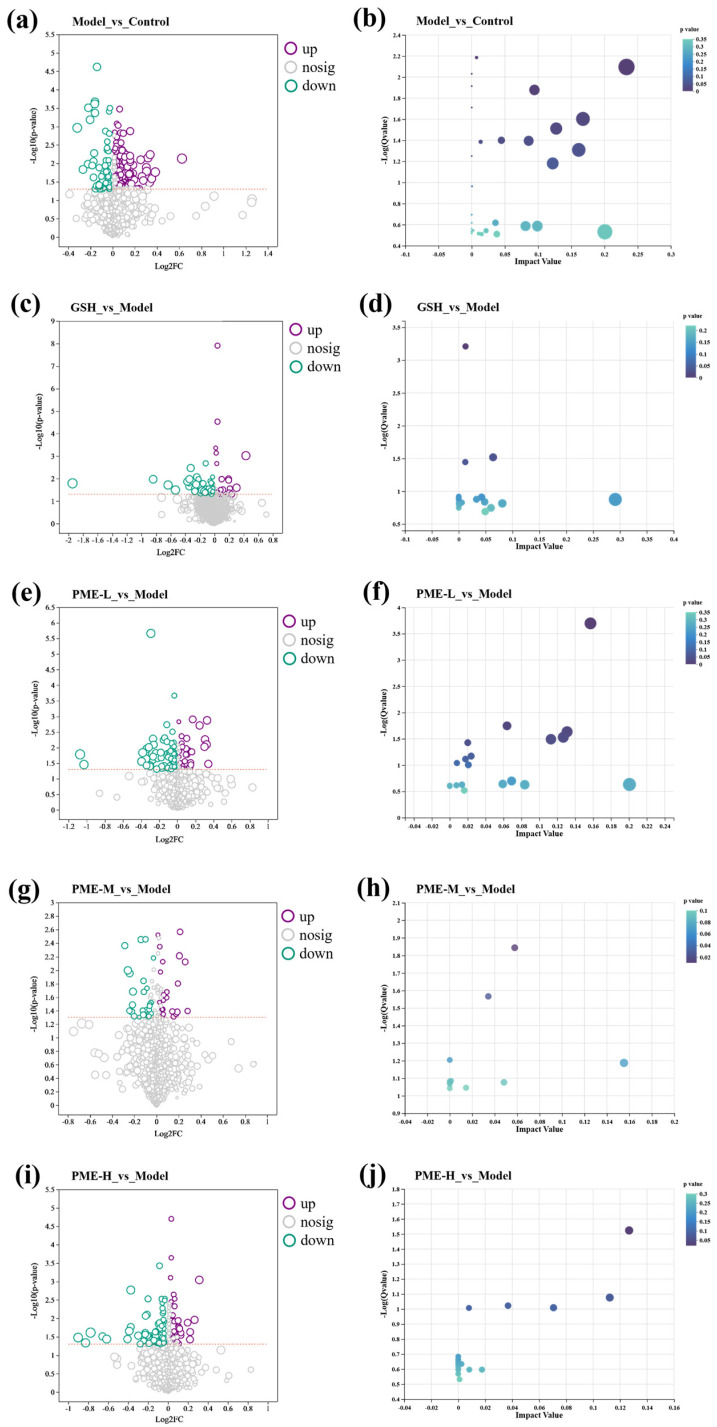
Differential metabolites and KEGG enrichment in different groups. Volcanic plot of differential metabolites between Model and Control groups (**a**), KEGG enrichment analysis between the Model and Control groups (**b**), volcanic plot of differential metabolites between GSH and Model groups (**c**), KEGG enrichment analysis between the GSH and Model groups (**d**), volcanic plot of differential metabolites between PME-L and Model groups (**e**), KEGG enrichment analysis between the PME-L and Model groups (**f**), volcanic plot of differential metabolites between PME-M and Model groups (**g**), KEGG enrichment analysis between the PME-M and Model groups (**h**), volcanic plot of differential metabolites between PME-H and Model groups (**i**), KEGG enrichment analysis between the PME-H and Model groups (**j**).

Differential metabolites were selected based on the criteria of FC > 1.0, *p* < 0.05, and VIP > 1. As shown in the figure, a total of 205 differential metabolites were identified between the Model and Control groups, with 141 upregulated and 64 downregulated in the Model group. Between the Model and treatment groups, 62, 113, 46, and 115 differential metabolites were identified in the GSH, PME-L, PME-M, and PME-H groups, respectively. Specifically, compared with the Model group, 20 metabolites were upregulated and 42 downregulated in GSH; 37 upregulated and 76 downregulated in PME-L; 23 upregulated and 26 downregulated in PME-M; and 37 upregulated and 78 downregulated in PME-H. KEGG pathway enrichment analysis was performed for the differential metabolites in each comparison group, and pathways with *p* < 0.05 were considered significantly enriched. As shown in Figure 10a,b, 205 differential metabolites were analyzed in the Model versus Control comparison, identifying 63 metabolic pathways, mainly including Tryptophan metabolism, Phenylalanine metabolism, Arginine and proline metabolism, Glycerophospholipid metabolism, Phenylalanine, tyrosine and tryptophan biosynthesis, Tyrosine metabolism, Pyrimidine metabolism, Ubiquinone and other terpenoid-quinone biosynthesis, Aminoacyl-tRNA biosynthesis, Valine, leucine and isoleucine biosynthesis, Valine, leucine and isoleucine degradation. In the GSH versus Model comparison (Figure 10c,d), 62 differential metabolites were analyzed, identifying 20 metabolic pathways, mainly including Nicotinate and nicotinamide metabolism and Glycine, serine and threonine metabolism. In the PME-L versus Model comparison (Figure 10e,f), 113 differential metabolites were analyzed, identifying 39 metabolic pathways, mainly including Pyrimidine metabolism and Tryptophan metabolism, Citrate cycle, Valine, leucine and isoleucine biosynthesis, Galactose metabolism, Pantothenate and CoA biosynthesis. In the PME-M versus Model comparison (Figure 10g,h), 49 differential metabolites were analyzed, identifying 22 metabolic pathways, mainly including Glycerophospholipid metabolism and Steroid hormone biosynthesis. In the PME-H versus Model comparison (Figure 10i,j), 115 differential metabolites were analyzed, identifying 27 metabolic pathways, mainly including Citrate cycle.

**Figure 11:**
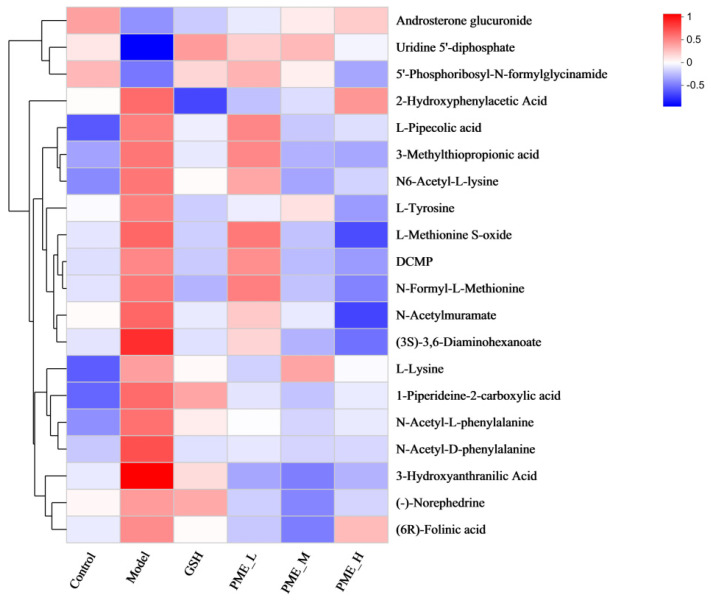
Characteristic metabolite analysis. The heatmap diagram of differential metabolites. Colors represent the relative intensities of each metabolite (red: high; blue: low).

To evaluate the metabolic changes induced by UVB radiation and the subsequent effects of PME treatment, a heatmap comprising 20 metabolites was constructed (Figure 11). UVB exposure elevated the levels of several amino acid-related metabolites, including N6-acetyl-L-lysine, L-tyrosine, L-methionine S-oxide, N-formyl-L-methionine, (3S)-3,6-diaminohexanoate, L-lysine, N-acetyl-L-phenylalanine, and N-acetyl-D-phenylalanine. Conversely, the levels of metabolites such as androsterone glucuronide, uridine 5′-diphosphate, and 5′-phosphoribosyl-N-formylglycinamide were decreased by UVB exposure. Importantly, PME treatment effectively counteracted these UVB-induced metabolic disturbances, suggesting its potential role in restoring metabolic homeostasis under UVB-induced alterations.

### 2.10. The Effects of PME on the Contents of SCFAs in Photoaged Nude Mice

**Figure 12:**
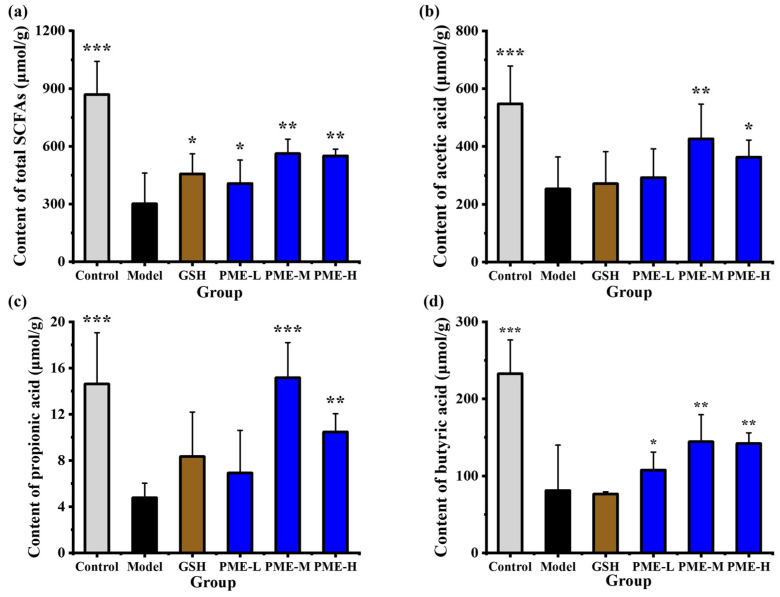
Effects of PME on Total SCFAs (**a**), Acetic acid (**b**), propionic acid (**c**) and butyric acid (**d**) in photoaged nude mice. * *p* < 0.05, ** *p* < 0.01 or *** *p* < 0.001 vs. Model group. Data are presented as the mean ± SD (*n* = 6).

Short-chain fatty acids (SCFAs) are key metabolites produced by the gut microbiota. They are saturated aliphatic organic acids containing one to six carbon atoms [53]. SCFAs are mainly generated by microbial fermentation of undigested plant-derived carbohydrates and, to a lesser extent, from dietary proteins and peptides [54,55] Acetate, propionate and butyrate are transported to the liver where they serve as substrates for energy metabolism [56]. In this study, SCFA levels were measured to evaluate the in vivo anti-photoaging effect of PME. As shown in Figure 12a, total SCFAs were markedly reduced in the Model group (301.34 μmol/g) compared with the Control (868.69 μmol/g, *p* < 0.05), indicating that UVB exposure disrupted intestinal microbial metabolism. PME-M and PME-H treatment significantly restored total SCFAs to 562.12 μmol/g and 550.32 μmol/g, respectively (*p* < 0.05). Analysis of individual SCFAs showed similar trends. Acetic acid (Figure 12b) decreased from 547.76 μmol/g in the Control to 252.87 μmol/g in the Model (*p* < 0.05); PME-M raised it to 426.36 μmol/g (*p* < 0.05). Propionic acid (Figure 12c) fell from 14.61 μmol/g in the Control to 4.78 μmol/g in the Model (*p* < 0.05), and PME-M increased it to 15.15 μmol/g (*p* < 0.05). Butyric acid (Figure 12d) declined from 232.66 μmol/g in the Control to 81.05 μmol/g in the Model (*p* < 0.05); PME-M and PME-H increased butyrate to 144.34 μmol/g and 142.04 μmol/g, respectively (*p* < 0.05).

Specifically, short-chain fatty acids are not only end products of microbial metabolism but also key signaling molecules that link the gut to peripheral tissues. Acetate, propionate and butyrate serve as energy substrates for colonocytes and help maintain epithelial barrier integrity [57]. Butyrate in particular can inhibit histone deacetylases and activate G-protein coupled receptors such as GPR41 and GPR43 (and GPR109A), thereby modulating inflammatory responses and promoting regulatory T cell differentiation [58]. Systemically, SCFAs can influence oxidative stress, host metabolism and the immune microenvironment of the skin, processes that are closely related to photoaging-mediated inflammation, collagen degradation and barrier dysfunction. These results suggested that PME effectively alleviated UVB-induced reductions in SCFA production, thereby potentially contributing to the restoration of gut microbial metabolic activity and intestinal homeostasis. PME-induced restoration of SCFA levels could reflect at least two mechanisms. It may provide fermentable substrates that fuel SCFA-producing bacteria, and it may modulate gut microbial composition to increase the abundance or activity of propionate- and butyrate-producing taxa.

### 2.11. The Effects of PME on Diversity and Composition of Gut Microbiota in Photoaged Nude Mice

**Figure 13:**
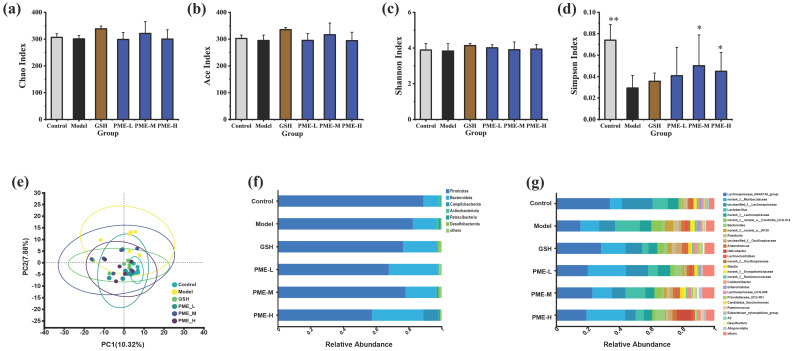
Effects of PME on Chao Index (**a**), Ace Index (**b**), Shannon Index (**c**), and Simpson Index (**d**); PCA analysis (**e**); PME modulates the relative abundance of gut microbiota on phylum level (**f**) and genus level (**g**). * *p* < 0.05, ** *p* < 0.01 vs. Model group. Data are presented as the mean ± SD (*n* = 6).

Alpha diversity was evaluated using the Chao, ACE, Shannon, and Simpson indexes. Chao and ACE primarily estimate species richness, whereas Shannon and Simpson capture aspects of richness and evenness [59]. As shown in Figure 13a–c, the Chao index, Ace index and Shannon index decreased in the Model group, although there was no significant difference compared with the Control group. After treatment with GSH and PME, the Chao index, Ace index and Shannon index were increased, but there was also no significant difference compared with the Model group. As shown in Figure 13d, the Simpson index decreased significantly (*p* < 0.05) in the Model group but increased after PME and GSH treatment, although no significant differences were observed compared with the Model group. These results suggested that UVB irradiation disrupted gut microbiota by decreasing evenness and potentially reducing richness, and PME treatment might attenuate this effect.

Beta diversity was assessed using PCA analysis (Figure 13e). Notably, the GSH, PME-L, and PME-H groups clustered closely with the Control group, indicating that these treatments positively modulate gut microbiota structure. Furthermore, alterations in gut microbial community structures were associated with significant changes in relative abundance patterns, observable at both the phylum and genus levels. As shown in Figure 13f, at the phylum level, the main species were *Firmicutes*, *Bacteroidota*, *Campilobacterota*, *Actinobacteriota*, *Patescibacteria*, *Desulfobacterota*, etc. Compared with the Control group, the abundance of *Firmicutes* was decreased and the abundance of *Bacteroidota* was increased in the Model group. UVB irradiation increased the abundance of *Patescibacteria* compared with the Control group, and GSH, PME-L, PME-H decreased the abundance of *Patescibacteria*. As shown in Figure 13g, at the genus level, the main species were *Lachnospiraceae_NK4A136_group*, *norank_f__Muribaculaceae*, *unclassified_f__Lachnospiraceae*, *Lactobacillus*, *norank_f__norank_o__Clostridia_UCG-014*, etc. Compared with the Control group, UVB irradiation decreased the abundance of the *Lachnospiraceae_NK4A136_group* and *unclassified_f_Lachnospiraceae*, while increasing the abundance of *norank_f_Muribaculaceae*, *Bacteroides*, *Lactobacillus*, *Roseburia*, *norank_f_norank_o_Clostridia_UCG-014*, and *norank_f_norank_o_RF39*. After intervention with PME and GSH, the abundance of the *Lachnospiraceae_NK4A136_group* and *unclassified_f_Lachnospiraceae* was increased, while the abundance of *norank_f_norank_o_Clostridia_UCG-014*, *Lactobacillus*, *norank_f_norank_o_RF39*, and *Roseburia* was reduced.

**Figure 14:**
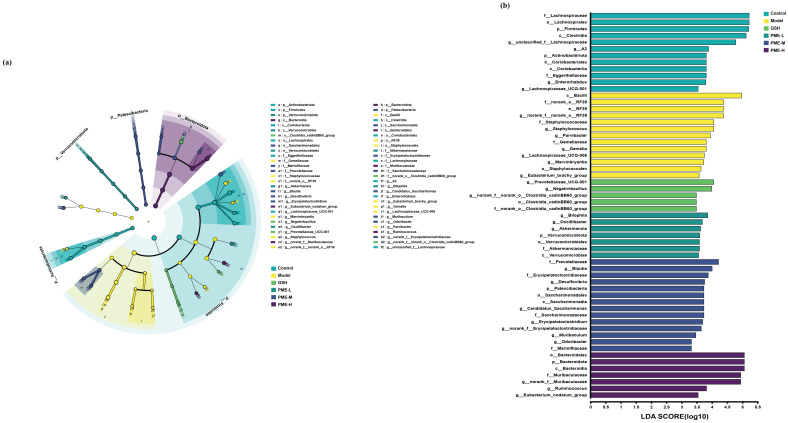
The species difference analysis after PME intervention. Cladogram of LEfSe analysis (**a**), distribution histogram (LDA > 3) (**b**).

LEfSe analysis was conducted with an LDA value of 3 to identify biomarkers that showed significant differences between groups. As shown in Figure 14a,b, the distinctly abundant bacterial taxa in the gut microbiota of the Control group mice were *Firmicutes* and *Actinobacteriota* at the phylum level, *Clostridia* and *Coriobacteriia* at the class level, *Lachnospirales* and *Coriobacteriales* at the order level, *Lachnospiraceae* and *Eggerthellaceae* at the family level, *unclassified_f__Lachnospiraceae*, *A2*, *Enterorhabdus* and *Lachnospiraceae_UCG-001* at the genus level. The distinctly abundant bacterial taxa in the gut microbiota of the Model group mice were *Bacilli* at the class level, *RF39* and *Staphylococcales* at the order level, *norank_o__RF39*, Staphylococcaceae and Gemellaceae at the family level, norank_f__norank_o__RF39, *Staphylococcus*, *Parvibacter*, *Gemella*, *Lachnospiraceae_UCG-006*, *Marvinbryantia* and *Eubacterium_brachy_group* at the genus level. The distinctly abundant bacterial taxa in the gut microbiota of the GSH group mice were *lostridia_vadinBB60_group* at the order level, *norank_o__Clostridia_vadinBB60_group* at the family level, *Prevotellaceae_UCG-001*, *Negativibacillus* and *norank_f__norank_o__Clostridia_vadinBB60_group* at the genus level. The distinctly abundant bacterial taxa in the gut microbiota of the PME-L group mice were *Verrucomicrobiota* at the phylum level, *Verrucomicrobiae* at the class level, *Verrucomicrobiales* at the order level, *Akkermansiaceae* at the family level, *Bilophila*, *Oscillibacter* and *Akkermansia* at the genus level. The distinctly abundant bacterial taxa in the gut microbiota of the PME-M group mice were *Patescibacteria* at the phylum level, *Saccharimonadia* at the class level, *Saccharimonadales* at the order level, *Prevotellaceae*, *Erysipelatoclostridiaceae*, *Saccharimonadaceae* and *Marinifilaceae* at the family level, *Blautia*, *Desulfovibrio*, *Candidatus_Saccharimonas*, *Erysipelatoclostridium*, *norank_f__Erysipelatoclostridiaceae*, *Muribaculum* and *Odoribacter* at the genus level. The distinctly abundant bacterial taxa in the gut microbiota of the PME-H group mice were *Bacteroidota* at the phylum level, *Bacteroidia* at the class level, *Bacteroidales* at the order level, *Muribaculaceae* at the family level, *orank_f__Muribaculaceae*, *Ruminococcus* and *Eubacterium_nodatum_group* at the genus level.

### 2.12. The Correlation Analysis

**Figure 15:**
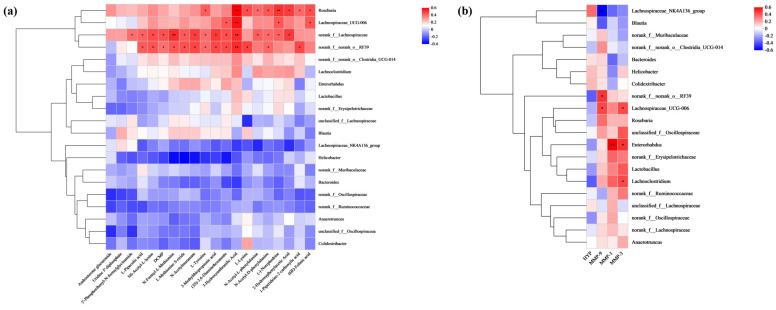
Correlation analysis. Correlation analysis between gut microbiota at genus level and differential metabolites (**a**), correlation analysis of gut microflora at genus level and skin photoaging indices (**b**). Blue denotes a negative correlation, and red indicates a positive correlation. The significant differences between the groups are indicated as * *p* < 0.05, ** *p* < 0.01.

Pearson’s correlation analysis of the 20 most abundant genera revealed multiple significant associations between gut taxa and serum metabolites. As shown in Figure 15a, norank_f__Lachnospiraceae and norank_f__norank_o__RF39 were significantly positively correlated with most metabolites, Roseburia were significantly positively correlated with (6R)-Folinic acid, 1-Piperideine-2-carboxylic acid, 2-Hydroxyphenylacetic Acid, (-)-Norephedrine, etc. Lachnospiraceae_UCG-006 was significantly positively correlated with (6R)-Folinic acid, (-)-Norephedrine, 3-Hydroxyanthranilic Acid and (3S)-3,6-Diaminohexanoate, indicating that the regulatory effects of PME on serum metabolites were associated with changes in the gut microbiota.

The association between the skin photoaging indices and the relative abundance at the genus level of gut microbiota (top 20) was analyzed (Figure 15b). The results showed that Lachnospiraceae_NK4A136_group was significantly negatively correlated with MMP-9, norank_f__norank_o__RF39 was significantly positively correlated with MMP-9, Lachnospiraceae_UCG-006 was significantly positively correlated with MMP-3 and MMP-9, Enterorhabdus was significantly positively correlated with MMP-1 and MMP-3, Lachnoclostridium was significantly positively correlated with MMP-3, indicating that these gut microbial species could contribute to alleviating UVB-induced symptoms.

A previous study supported the existence of a gut–skin axis, whereby the gut microbiota modulate skin homeostasis through immune and metabolic pathways [60]. Dysbiosis was reported to be associated with various skin disorders [61], and modulation of the gut microbiota was therefore proposed as a promising strategy for skin protection [62]. Notably, small bioactive peptides could cross the intestinal barrier, enter systemic circulation, and distribute to peripheral tissues, including the skin, where they might exert direct effects on skin cells or indirectly attenuate oxidative stress and inflammation via immune regulation [5]. For instance, Oesser et al. demonstrated that more than 90% of orally administered ^14^C-labeled gelatin hydrolysate was absorbed within 6 h in mice and subsequently transported to the skin, where it exhibited biological activity [63]. Collectively, these findings suggested the potential of PME to exert systemic protective effects against photoaging through modulation of the gut–skin axis.

## 3. Materials and Methods

### 3.1. Materials and Reagents

*Pinctada martensii* meat was obtained from Ronghui Co., Ltd. (Zhanjiang, China). As described previously [64], briefly, 100 g of *P. martensii* meat was chopped, homogenized with 200 mL distilled water, and adjusted to pH 3. The mixture was hydrolyzed with pepsin (Sigma-Aldrich, Burlington, MA, USA, 2000 U/mL) at 37 °C for 2 h, then pepsin was inactivated by adjusting the pH to 7.5. Subsequently, trypsin (Yuanye Bio-Technology Co., Ltd., Shanghai, China; 100 U/mL) and chymotrypsin (Macklin Biochemical Co., Ltd., Shanghai, China; 25 U/mL) were added and hydrolyzed at 37 °C for 2 h. Enzymes were inactivated by heating, the mixture was centrifuged, and the supernatant was freeze-dried to obtain PME.

A commercial kit for hydroxyproline (HYP) determination was purchased from Nanjing Jiancheng Bioengineering Institute (Nanjing, China). The kits for determining MMP-1, MMP-3 and MMP-9 were purchased from Solarbio Science & Technology Co., Ltd. (Beijing, China). The bicinchoninic acid (BCA) protein quantitation assay kit was purchased from KeyGenBio (Nanjing, China). All other chemicals and reagents used were of analytical grade.

### 3.2. Animals and Treatments

Sixty female BALB/c nude mice (8 weeks old, weight 18–22 g) were purchased from the Zhuhai Bestest Biotechnology Co., Ltd. (Zhuhai, China) and housed under specific pathogen-free (SPF) conditions. The animals were maintained in a controlled environment at 23–27 °C and 55–70% relative humidity, under a strict light–dark cycle, with ad libitum access to food and water. Prior to experimentation, the mice were acclimatized for 7 days. All experimental procedures and protocols were reviewed and approved by the South China Agricultural University Committee of Animal Experiments (approval ID: 2022B093).

Mice were randomly assigned to 6 groups: Control group (treated with saline), Model group (treated with saline), GSH group (treated with 150 mg/kg/day of GSH), PME-L group (treated with 150 mg/kg/day of PME), PME-M group (treated with 300 mg/kg/day of PME), and PME-H group (treated with 600 mg/kg/day of PME). All treatments were administered once daily by oral gavage. From week 1, all groups except Control were exposed to UVB irradiation four times per week (Monday, Wednesday, Friday, and Sunday) for five weeks using a HOPE-MED 8140 photoaging device (Tianjin Hepu Industry & Trade Co., Tianjin, China). UVB irradiation was delivered using lamps emitting at 313 nm, positioned 30 cm above the dorsal skin of the mice. During irradiation, mice were restrained in custom-designed holders to expose the dorsal skin. The irradiance was monitored throughout the experiment using a UV light meter (UV-340A, Luchang Electronic Enterprise, Taipei, Taiwan). The UVB dose was gradually increased from 200 mJ/cm^2^ during weeks 1–2, to 300 mJ/cm^2^ during weeks 2–3, and finally to 400 mJ/cm^2^ during weeks 3–6. Body weights were recorded daily. At the end of the experiment, six mice per group were humanely euthanized, followed by cervical dislocation to ensure death, in accordance with institutional animal care guidelines., and the dorsal skin of each mouse was photographed for macroscopic evaluation. Intestinal contents, organs, blood, and dorsal skin samples were then collected from each mouse and stored at −80 °C for further analysis.

### 3.3. Organ Index

After sacrificing the mice, the heart, liver, spleen, lung, kidney, and total body were collected and weighed. Organ indices were subsequently calculated using the following formula:

organ index (mg/g) = weight of the organ (mg)/body weight (g).(1)

### 3.4. Histopathology

#### 3.4.1. Tissue Paraffin Embedding and Sectioning

The isolated dorsal skin tissues were fixed in 4% paraformaldehyde at room temperature for 24 h, dehydrated through a graded ethanol series, cleared in xylene, and embedded in paraffin at 65 °C. Paraffin blocks were sectioned at a thickness of 4 µm using the paraffin microtome (Leica, Wetzlar, Germany), the sections were floated on 40 °C water, mounted onto adhesive slides, baked at 60 °C, and stored at room temperature for further use. 

#### 3.4.2. Hematoxylin and Eosin (HE) Staining

Skin tissues from mice were fixed in formalin, embedded in paraffin, and cut into 4 μm sections. After deparaffinization, sections were incubated with a high-definition staining pretreatment solution for 1 min, stained with hematoxylin for 3–5 min, differentiated with a hematoxylin differentiation solution, blued with a hematoxylin bluing solution, and counterstained with eosin for 15 s (Servicebio, Wuhan, China). Sections were then dehydrated in 100% ethanol, cleared in xylene, and mounted with neutral balsam. Nuclei appeared blue and cytoplasm pink to red under a bright-field microscope. Images were quantified using ImageJ v1.54f (National Institutes of Health, Bethesda, MD, USA) software.

#### 3.4.3. Masson’s Trichrome Staining

Skin tissues from mice were fixed in formalin, embedded in paraffin, and cut into 4 μm sections. After deparaffinization, sections were stained using a Masson staining kit (Servicebio, Wuhan, China), rinsed with 1% glacial acetic acid, dehydrated in 100% ethanol, cleared in xylene, and mounted with neutral balsam. Under a bright-field microscope, collagen fibers stained blue, whereas muscle fibers, cytoplasm, and red blood cells stained red. Images were quantified using ImageJ v1.54f (NIH, MD, USA) software.

#### 3.4.4. Sirius Red Staining

Skin tissues from mice were fixed in formalin, embedded in paraffin, and cut into 4 μm sections. After deparaffinization, sections were stained with Sirius Red staining solution for 8 min (Servicebio, Wuhan, China), dehydrated in 100% ethanol, cleared with xylene, and mounted with neutral balsam. Under a polarized microscope, collagen type I appeared bright orange-red, whereas type III appeared green. Images were analyzed using ImageJ v1.54f (NIH, MD, USA) with the CMM_PRBRF_Analyser macro [65].

### 3.5. Immunofluorescence Staining

Dorsal skin samples were collected and fixed in 4% paraformaldehyde for 24 h. Following paraffin embedding, 4 µm tissue sections were deparaffinized in xylene and rehydrated through a graded ethanol series. For antigen retrieval, slides were incubated in EDTA buffer (pH 8.0) and washed three times with PBS (pH 7.4). Sections were blocked with 3% BSA for 30 min to prevent nonspecific binding. Primary antibodies were applied at the indicated dilutions and incubated overnight at 4 °C. After three PBS washes, sections were incubated with secondary antibodies for 50 min at room temperature in a light-protected humid chamber. Following three washes with PBS, the sections were stained with DAPI for 15 min and imaged on an immunofluorescence microscope (Nikon, Tokyo, Japan). Image acquisition used standardized exposure settings, and fluorescence intensity was quantified with ImageJ v1.54f (NIH, MD, USA).

### 3.6. Determination of HYP, MMP-1, MMP-3 and MMP-9 in the Skin

Skin tissue samples were placed in enzyme-free homogenization tubes and homogenized in pre-chilled PBS using a tissue grinder at −10 °C and 60 Hz. Homogenates were centrifuged at 12,000× *g* for 20 min, and the supernatants were collected for subsequent analyses. The HYP, MMP-1, MMP-3, and MMP-9 levels were measured using commercial ELISA kits according to the manufacturer’s instructions. Concentrations of MMP-1, MMP-3, and MMP-9 were normalized to total protein using a BCA assay kit.

### 3.7. Determination of Short-Chain Fatty Acids (SCFAs)

SCFAs in colon contents were determined as described by Chen et al. [66]. Briefly, 30–120 mg of colon contents were mixed with 1 mL of distilled water and vortexed thoroughly. Samples were centrifuged at 12,000× *g* for 5 min at 4 °C, and the supernatant was filtered through a 0.22 μm membrane. SCFA levels (acetic, propionic, and butyric acids) were measured by 7820A gas chromatography (Agilent, Santa Clara, CA, USA) using an AE-FFAP capillary column (30 m × 0.25 mm × 0.25 μm; Atech Technologies, Lanzhou, China) and a hydrogen flame detector. Injection temperature was 240 °C, detector temperature 250 °C, and injection volume was 0.3 μL. Nitrogen (N2) was used as the carrier gas, and samples were analyzed by split injection (split ratio 2:1) with a carrier gas flow rate of 1 mL/min. The oven program was 100 °C for 0.5 min, ramp at 4 °C/min to 160 °C, then ramp at 10 °C/min to 220 °C and hold at 220 °C for 2 min. SCFA concentrations were calculated from standard curves and normalized to the wet weight of the original colon-content sample.

### 3.8. 16S rRNA Gut Microbiota Profiling

Cecal contents were snap-frozen in liquid nitrogen and stored at −80 °C until use. Microbial community genomic DNA was extracted using the E.Z.N.A.^®^ soil DNA Kit (Omega Bio-tek, Norcross, GA, USA). The V3-V4 hypervariable region of the bacterial 16S rRNA gene was amplified with primer 338F (5′-ACTCCTACGGGAGGCAGCAG-3′) and 806R (5′-GGACTACHVGGGTWTCTAAT-3′) by an ABI GeneAmp^®^ 9700 PCR thermocycler (Applied Biosystems, Carlsbad, CA, USA). Purified amplicons were pooled in equimolar and paired-end sequencing on an Illumina MiSeq PE300 platform/NovaSeq PE250 platform (Illumina, San Diego, CA, USA).

The raw 16S rRNA gene sequencing reads were demultiplexed and quality-filtered using fastp (version 0.20.0). Subsequently, the reads were merged using FLASH (version 1.2.7) [67]. Operational taxonomic units (OTUs) were clustered at 97% similarity using UPARSE v7.1 [68,69], with chimeric sequences identified and removed. Taxonomy for each OTU representative sequence was assigned using the RDP Classifier (version 2.2) [70] against a 16S rRNA reference database with a confidence threshold of 0.7. Taxonomic assignments were made at the phylum, class, order, family, and genus levels. Species composition and differential-abundance analyses were performed in R software (version 3.3.1). The α diversity was analyzed with mothur (version v.1.30.2). β diversity was conducted using Qiime (1.9.1) and R software (version 3.3.1).

### 3.9. Untargeted Metabolomics Analysis

Blood was collected from mice via the retro-orbital venous plexus, allowed to clot at 4 °C, and centrifuged at 3000× *g* for 10 min at 4 °C. The serum supernatant was collected, filtered through a 0.22 μm membrane, and submitted to Majorbio Bio-Pharm Technology Co., Ltd. (Shanghai, China) for untargeted LC-MS/MS analysis. Briefly, chromatographic separation was performed on a Waters BEH C18 column (100 × 2.1 mm i.d., 1.8 μm). Analyses were carried out on a quadrupole time-of-flight mass spectrometer (TripleTOF 5600+, AB Sciex, Framingham, MA, USA) with an electrospray ionization (ESI) source in both positive and negative ion modes. Mobile phases were solvent A, 0.1% formic acid in water; solvent B, 0.1% formic acid in acetonitrile:isopropanol (1:1, *v*/*v*). Mass spectra were acquired across *m*/*z* 50–1000. Instrument settings included ion spray voltage +5000 V (positive) and −4000 V (negative), declustering voltage 80 V, curtain gas 30 psi, nebulizer/spray gas 50 psi, auxiliary/drying gas 50 psi, ion source temperature 500 °C, and collision energy cycled between 20 and 60 eV.

Metabolite annotation was performed by matching MS/MS spectra to HMDB (http://www.hmdb.ca/, accessed on 5 December 2022), METLIN (https://metlin.scripps.edu/, accessed on 5 December 2022), and Majorbio (https://cloud.majorbio.com, accessed on 5 December 2022) databases. Differential metabolites were selected using variable importance in projection (VIP) > 1 from multivariate models and *p* < 0.05 from univariate testing. Data processing and statistical analyses were performed on the Majorbio Cloud platform. Pathway enrichment and metabolic pathway analyses were conducted based on the KEGG database.

### 3.10. Statistical Analysis

Statistical comparisons among groups were performed by one-way analysis of variance (ANOVA) followed by Tukey’s post hoc test. Differences were considered significant at a *p* value of 0.05. Statistical analyses were performed using the SPSS 26 software (Chicago, IL, USA). Metabolomics and gut-microbiota data were processed on the Majorbio Cloud Platform (https://cloud.majorbio.com, accessed on 5 December 2022).

## 4. Conclusions

Oral supplement of PME effectively attenuated UVB-induced skin photoaging in BALB/c nude mice. PME markedly reduced UVB-induced epidermal hyperplasia and collagen fiber fragmentation, restored dermal collagen content and the type I/type III collagen ratio, and significantly suppressed MMP-1, MMP-3 and MMP-9 levels, likely through inhibition of UVB-activated ERK signaling, thereby limiting extracellular matrix degradation. At the systemic level, PME shifted serum metabolomic profiles closer to those of non-irradiated controls, restored colonic SCFAs concentrations, and partially reversed UVB-induced gut dysbiosis, including recovery of several Lachnospiraceae taxa. Correlation analyses further support a gut microbiota–metabolite–skin axis linking PME-induced microbial and metabolic changes to improvements in skin photoaging markers. Together, these results indicate that PME exerts multi-protective effects against UVB photoaging, involving inhibition of matrix degrading enzymes, and probable gut-microbiota-metabolite-mediated contributions.

## Data Availability

The original contributions presented in this study are included in the article. Further inquiries can be directed to the corresponding author.
